# Realist trials and the testing of context-mechanism-outcome configurations: a response to Van Belle et al.

**DOI:** 10.1186/s13063-016-1613-9

**Published:** 2016-10-01

**Authors:** Chris Bonell, Emily Warren, Adam Fletcher, Russell Viner

**Affiliations:** 1 London School of Hygiene & Tropical Medicine, 15-17 Tavistock Place, London, WC1H 9SH UK; 2 Cardiff University, Glamorgan Building, King Edward VII, Cardiff, CF10 3WT UK; 3 University College London Institute of Child Health, 30 Guilford Street, London, WC1N 1EH UK

**Keywords:** Randomized controlled trials, Realist evaluation, Scientific realism, Causation

## Abstract

**Background:**

Van Belle et al. argue that our attempt to pursue realist evaluation via a randomised trial will be fruitless because we misunderstand realist ontology (confusing intervention mechanisms with intervention activities and with statistical mediation analyses) and because RCTs cannot comprehensively examine how and why outcome patterns are caused by mechanisms triggered in specific contexts.

**Methods:**

Through further consideration of our trial methods, we explain more fully how we believe complex social interventions work and what realist evaluation should aim to do within a trial.

**Results:**

Like other realists, those undertaking realist trials assume that: social interventions provide resources which local actors may draw on in actions that can trigger mechanisms; these mechanisms may interact with contextual factors to generate outcomes; and data in the ‘empirical’ realm can be used to test hypotheses about mechanisms in the ‘real’ realm. Whether or not there is sufficient contextual diversity to test such hypotheses is a contingent not a necessary feature of trials. Previous exemplars of realist evaluation have compared empirical data from intervention and control groups to test hypotheses about real mechanisms. There is no inevitable reason why randomised trials should not also be able to do so. Random allocation merely ensures the comparability of such groups without necessarily causing evaluation to lapse from a realist into a ‘positivist’ or ‘post-positivist’ paradigm.

**Conclusions:**

Realist trials are ontologically and epistemologically plausible. Further work is required to assess whether they are feasible and useful but such work should not be halted on spurious philosophical grounds.

## Background

Van Belle et al. argue that our attempt to pursue realist evaluation via a randomised controlled trial (RCT) will be fruitless first, because we misunderstand realist ontology (confusing intervention mechanisms with intervention activities and with statistical mediation analyses) and second, because RCTs cannot comprehensively examine how and why outcome patterns are caused by mechanisms triggered in specific contexts [1]. We have found our ongoing debate with realist evaluators extremely useful in clarifying our thinking. In response to Van Belle et al.’s critique, we attempt to explain more fully how we believe complex social interventions work and what realist evaluation should aim to do within a trial. Finally, we counter the argument that RCTs are inimical to realist enquiry.

## Main text

### A realist understanding of interventions and mechanisms

Let us first be clear about the nature of the ‘Learning Together’ intervention referred to in our earlier article [2] and how it is meant to work. We completely agree with Van Belle et al. that interventions comprise a series of resources. Learning Together aims to reduce bullying and aggression in secondary schools by providing schools with the following resources: (1) lesson plans and slides for a social and emotional skills curriculum; (2) a report of data on local student needs, a manual and an external facilitator; and (3) training sessions for staff on restorative practice. We offer these resources to schools with the hope that staff and students decide to use them in order to facilitate various actions such as: lessons on social/emotional skills; revisions to school policies and other locally decided school-level decisions; and restorative practice sessions in which staff and students respond to incidents of aggression and bullying. We theorise that through these actions various mechanisms may be triggered. We agree with Van Belle et al. that social interventions cannot introduce mechanisms directly but only via a process of participants acting on the resources provided. In the case of Learning Together, there are various intended mechanisms all involving the erosion of various ‘boundaries’ between and among staff and students, and between students’ academic and broader development. We theorise that these boundaries will be eroded not by the intervention directly but via staff and students engaging in actions, supported by the intervention resources, to enhance relationships across the school and by reorienting school activities to focus on students’ holistic development, which should include but not be limited to academic attainment.

Based on the theory of human functioning and school organisation [3] as well as on qualitative research on the school environment and young people’s health [4], we theorised in our original article that the erosion of these boundaries will encourage more students, especially those from working class backgrounds, to feel committed to school, less committed to anti-school peer groups and less engaged in practices which go against school formal rules and informal norms, including bullying and aggression [2]. We further theorised that these mechanisms will be triggered and play out differently in different contexts. For example, we theorised that in schools where staff already give some priority to promoting students’ overall wellbeing, the intervention activities are more likely to be implemented, so the intervention mechanisms, particularly those concerning the erosion of boundaries between students’ academic and broader development, are more likely to be triggered. And we theorised that in schools with more working class students, the erosion of the boundaries will lead to a higher proportion of students becoming committed to school (because we theorise that the aforementioned boundaries in particular hamper the educational engagement of working class students). We hope this fuller description of our intervention and its mechanisms reassures readers that our understanding of the Learning Together intervention is compatible with realist ontology and realist evaluation practice.

#### A realist understanding of empirical evaluation

We continue to contend that our approach to research in general and evaluation in particular, is realist in orientation. Realists suggest there is an ‘empirical’ realm consisting of the data researchers collect and analyse. This empirical realm provides a window, albeit an indirect one, on an ‘actual’ realm of occurrences apparent to participants. This actual realm in turn reflects a ‘real’ realm made up of structural mechanisms which are unobservable but which are the causes of the actual and empirical realms [5]. In terms of epistemology, realists believe they can identify objective truths describing the actual realm and can uncover the true causal mechanisms of the real realm based upon data from the empirical realm. Like Van Belle et al., we believe that even if intervention mechanisms do occur, they will not be directly observable. Boundaries and commitment to school lie in the realm of the real; they cause observable phenomena but are not themselves observable.

The activities our intervention is intended to promote are, in critical realist parlance, in the actual realm, as are the outcomes we hope will arise as a result of the intervention. Staff and students will be able to observe intervention activities, such as restorative sessions, as well as the behaviours, such as bullying, that the intervention is aiming to reduce. However, the data collected in the course of the RCT of Learning Together (like data collected in any form of research) are not a direct and unproblematic window into this actual realm. Our outcome evaluation is not collecting data on bullying or aggression directly. Rather, it is collecting data on student answers to questionnaires asking about their experiences of these practices. Similarly, our process evaluation is not collecting data on activities such as policy review and restorative sessions directly. Rather, it collects data in the form of notes that researchers make when they observe these sessions or in the form of the accounts of teachers or students when they are interviewed about their experiences of these activities. In critical realist terms, these data are in the realm of the empirical. We appreciate that all sorts of factors might mean that these data do not provide a full or unproblematic representation of events in the realm of the actual. Nonetheless, they should provide some guide as to what is happening.

Based on our theorising about how mechanisms (in the realm of the real) interact with context to produce outcomes (in the realm of the actual), we have hypothesised that in statistical analyses of outcome indicators (in the realm of the empirical), students in the schools randomly selected to implement the intervention will report less bullying and aggression than students in schools randomly selected to be controls [2]. We also hypothesised that in mediation analyses, the association between trial arm and the empirical indicators of bullying and aggression will be reduced by adjustment for indicators of increased student commitment to school. And we further hypothesised that in statistical moderation analyses, baseline school-level aggregate indicators of staff priorities and school-level indicators of student-reported socio-economic status will moderate the association found between intervention arm and our measures of bullying and aggression.

The above are examples of how we intend to use statistical analyses (in the realm of the empirical) to test hypotheses about how, in the realm of the real, context and mechanisms interact to generate outcomes (known as context-mechanism-outcome or ‘CMO configurations’). Van Belle et al. say very little about why realist RCTs are unlikely to be able to empirically test hypotheses about CMO. They assert, without reference to evidence or further argument, that “Given the need for randomisation and control in an RCT, only relatively few and simple CMO configurations can be tested at a time.” We disagree. We aim to undertake analyses to test multiple CMOs including but not limited to those above. Some of these are based a priori on theory while others have been and will continue to be developed based on qualitative research within the trial. Testing these should be perfectly possible within an RCT. These analyses should help us gain a more vivid and nuanced understanding of how and why reductions in bullying and aggression are caused by mechanisms of boundary erosion and school engagement which are triggered and play out differently in diverse school contexts. But whether this is the case or not is ultimately an empirical question. It cannot be judged until we complete our analyses. We see nothing about random allocation which impede such analyses. Indeed, doing such analyses within an RCT has the crucial advantage of control of confounding. This is so important in public health because even important interventions will often have quite small effects for each individual participant, which can be impossible to distinguish from other confounding influences [6]. We agree that our ability to assess CMO configurations would be undermined if trials contained insufficient variety in characteristics of place or person because of excessively tight inclusion criteria for sampling clusters or individuals. But while this may sometimes occur in trials, it is not a necessary feature, particularly of pragmatic effectiveness trials. The other obvious impediments to the proposed analyses are measurement error and lack of statistical power. These are real challenges but they are in no way necessary or particular features of trials as opposed to any other designs.

Furthermore, we would like to stress that developing and empirically testing such hypotheses does not mean, as Van Belle et al. seem to suggest, that we are confusing the empirical with the real, reducing the causal mechanism of our intervention to statistical mediation analyses or reducing the context of the intervention to statistical moderation analyses. We are using crude and indirect quantitative data (which exist in the realm of the empirical) as a way of indirectly testing whether our theories about mechanisms (which exist in the realm of the real) might be correct. Moreover, we are also using qualitative research to deepen our understanding of how mechanisms might work and using this to refine our theories and hypotheses. Like our quantitative research, our qualitative research examines empirical data (this time in the form of student and staff accounts) as an indirect window on the actual (participants’ experiences) and the real (how mechanisms unfold through interactions between individual agency and social structures) realms.

And if our analyses do not support the above hypotheses, this will not mean we immediately conclude that the theorized mechanisms do not exist. Null results could indicate that the context causes the mechanisms to remain unactivated (for example, schools do not implement the intervention or implementation fails to trigger an erosion of boundaries) or the mechanism is activated but counteracted by other mechanisms (for example, government initiatives cause schools to buttress the boundaries between students’ academic and broader development). We should stress that this approach is consistent with existing philosophy about how to interpret null results from RCTs of social interventions [7].

#### What is so bad about randomisation?

Although we think randomisation is extremely important technically in enabling us to assess statistical associations while minimizing bias, we think randomisation is philosophically trivial. We disagree that the use of randomisation will inevitably lead to our research lapsing from a realist into a positivist or post-positivist paradigm [8]. Randomisation ensures that the different groups we compare resemble each other as closely as possible in terms of all the characteristics (except from exposure to intervention resources) likely to affect outcomes, whether we know about these in advance or not. If we were to evaluate Learning Together by comparing rates of reported bullying and aggression between schools which chose to implement the intervention versus those which did not, there is a very strong likelihood that any reductions in indicators of bullying and aggression would wholly or partly (we would not know which) reflect baseline differences in the institutions and/or the individuals within them. Randomisation is merely a practical tool to reduce confounding. It does not fundamentally change the nature of the way we view or research the social world, or affect how we will use comparative empirical data to test hypotheses about mechanisms.

Proponents of non-randomised realist evaluation in fact often refer to the use of quantitative empirical data from different groups as exemplars of how to test hypotheses about intervention mechanisms. For example, Pawson and Tilley refer to an evaluation of prisoner education as a means of reducing recidivism, in which evaluators compared rates of reoffending in intervention sites with expected rates. The latter were generated from historical ‘usual treatment’ data. Van Belle et al. do not attempt to explain why such non-random comparisons of quantitative data are legitimately realist whereas our comparisons of data from randomly allocated groups are not [9].

It is true that the originator of critical realism objected to the use of RCTs in social as opposed to natural scientific enquiry [10] but this betrayed his misunderstanding of how natural and social scientific research is done. Bhaskar argued that experimental manipulation is used to create closed systems to neutralise external forces and so isolate the mechanisms being tested. He suggests that experimental manipulation in social science is impossible because social systems are open [5]. We agree that experiments in the physical sciences do often try to control and isolate causal factors. However, experiments in the biological sciences often take a different approach, because in biology many systems (such as ecosystems and bodily systems) cannot be closed. Many RCTs in the fields of environmental science and clinical pharmacology do not use randomisation to remove all other mechanisms in order to isolate the mechanism under investigation. Rather, randomisation is used, in effect, to hold these other mechanisms constant so that the mechanism under investigation can be viewed in their full context, i.e. to understand its impact alongside these other mechanisms. In other words, these trials measure ‘added value’ not ‘separated value’. The same principle applies to RCTs of social interventions. And in fact the same principle also applies to non-randomised comparative evaluations such as the realist review of prisoner education mentioned above. These designs use comparison groups to look for the added value of the intervention mechanisms against the backdrop of other influential mechanisms. The fact that these comparison groups are not assembled via random allocation does not alter this fact.

We would strongly agree with realists that RCTs are quite impractical for examining many questions in the social sciences - including perhaps the most interesting and important questions, such as what mechanisms account for the maintenance of class inequality [11] or secular reductions in violence [12]. It would obviously be impractical to randomly allocate people to different social classes or historical eras. And we would also acknowledge that for many of the most important public health interventions, control groups and/or random allocation are impractical, for example in the evaluation of the health impacts of smoking bans, alcohol taxes or seatbelt enforcement [13]. But in other cases, it is perfectly feasible to use RCTs to investigate public health interventions and, in such cases, randomised designs should not be rejected based on dogma.

Some evaluations of social interventions draw on naturally occurring random allocation. For example, in the USA researchers have examined whether educational attainment is higher in charter schools than community schools [14]. Here, evaluation is facilitated by the fact that some oversubscribed charter schools determine entry by random ballot. The evaluations done so far have not been realist and have not assessed CMO configurations. But it is possible to imagine that naturally randomised experiments could assess how context interact with mechanisms to generate outcomes. Would the opponents of realist trials argue that in order to do so, evaluators would have to find other, non-random comparisons to avoid the taint of positivism?

## Conclusions

Realist evaluation focuses on developing, refining and testing theories about how interventions provide resources which participants use to trigger mechanisms that interact with context to generate outcomes. This is extremely useful both in emphasising that evaluation studies should focus on the testing and refinement of intervention theory (rather than merely accrediting particular interventions as effective or not). It is also extremely helpful in providing a basis for understanding the importance of context, and for drawing on empirical evidence to consider how context might affect the implementation and effects of interventions in new settings. Realist evaluation has sometimes been described as methods neutral [15] and social science more generally as methodologically pluralist [16]. Indeed, Van Belle et al. state that realist evaluation should use “whatever data and analytic methods [are] appropriate to build, support, refute or refine plausible explanations that incorporate intervention, actors, outcomes, context and mechanisms”. We hope to persuade realists that RCTs have a place in this analytic panoply.

## Abbreviations

CMO: Context-mechanism-outcome
RCT: Randomised controlled trials
